# G-Protein-Coupled Receptor (GPCR) Signaling and Pharmacology in Metabolism: Physiology, Mechanisms, and Therapeutic Potential

**DOI:** 10.3390/biom15020291

**Published:** 2025-02-15

**Authors:** Yun Yeong Cho, Soyeon Kim, Pankyung Kim, Min Jeong Jo, Song-E Park, Yiju Choi, Su Myung Jung, Hye Jin Kang

**Affiliations:** 1 Department of Biotechnology, College of Life Science and Biotechnology, Yonsei University, Seoul 03722, Republic of Korea; yunie1116@naver.com (Y.Y.C.); pandol02@yonsei.ac.kr (P.K.); anniec@naver.com (M.J.J.); thddl4278@naver.com (S.-E.P.); 2 Department of Biological Sciences, Sungkyunkwan University (SKKU), Suwon 16419, Republic of Korea; soyeonkim98@skku.edu (S.K.); 2wixx@skku.edu (Y.C.)

**Keywords:** GPCR (g-protein coupled receptor), GPR40, GPR120, GLP-1R, ADRB1, ADRB2, ADRB3, metabolic disease

## Abstract

G-protein coupled receptors (GPCRs), the largest family of integral membrane proteins, enable cells to sense and appropriately respond to the environment through mediating extracellular signaling to intercellular messenger molecules. GPCRs’ pairing with a diverse array of G protein subunits and related downstream secondary messengers, combined with their ligand versatility-from conventional peptide hormone to numerous bioactive metabolites, allow GPCRs to comprehensively regulate metabolism and physiology. Consequently, GPCRs have garnered significant attention for their therapeutic potential in metabolic diseases. This review focuses on six GPCRs, GPR40, GPR120, GLP-1R, and ß-adrenergic receptors (ADRB1, ADRB2, and ADRB3), with GLP-1R recognized as a prominent regulator of system-level metabolism, while the roles of GPR40, GPR120 and ß-adrenergic receptors in central carbon metabolism and energy homeostasis are increasingly appreciated. Here, we discuss their physiological functions in metabolism, the current pharmacological landscape, and the intricacies of their signaling pathways via G protein and ß-arrestin activation. Additionally, we discuss the limitations of existing GPCR-targeted strategies for treating metabolic diseases and offer insights into future perspectives for advancing GPCR pharmacology.

## 1. Introduction

Obesity and associated chronic metabolic diseases, including type 2 diabetes, have emerged as critical global health concerns. These pathophysiologies are driven by imbalanced energy metabolism resulting from high-calorie intake, decreased physical activity—exacerbated during the recent COVID-19 pandemic—and increased life span as aging predisposes individuals to metabolic disorders [1,2,3]. Type 2 diabetes is characterized by insulin resistance and pancreatic β-cell failure [4,5]. During insulin resistance, the body’s tissues become unresponsive to insulin’s effects despite its production, while β-cell dysfunction impairs the pancreas’s ability to secrete sufficient insulin [6,7,8,9,10,11]. Since obesity exacerbates both these mechanisms, the global increase in obesity rates is considered a major contributor to the growing prevalence of type 2 diabetes [11,12]. Understanding the molecular mechanisms underlying obesity and its progression to type 2 diabetes is, therefore, crucial for developing effective prevention and treatment strategies. By uncovering the pathways linking obesity to insulin resistance and β-cell dysfunction, researchers can identify potential therapeutic targets to combat these interconnected diseases.

Given their central roles in cellular communication and metabolic regulation, G-protein coupled receptors (GPCRs) have emerged as key players in the pathophysiology of obesity and type 2 diabetes. GPCRs, also known as seven transmembrane receptors, are integral membrane proteins expressed on nearly all cell types throughout the body, translating extracellular signals into intracellular responses. GPCR activation typically involves an agonist binding to the receptor, stabilizing its conformation to recruit and activate intracellular signaling transducers [11,13,14,15]. GPCRs respond to a wide range of agonists, extending beyond classic peptide hormones (e.g., glucagon and GLP1) to include endogenous and exogenous metabolites such as sugars, free fatty acids, nucleotides, and microbial products, as well as synthetic agents [16,17,18,19]. Additionally, not only does each group of GPCR utilize distinct G-protein subunits that couple to specific second messenger pathways [20,21,22,23,24], but even the same receptor can activate different G-protein subtypes or β-arrestin signaling in a ligand-dependent manner. These features collectively make GPCR signaling highly pleiotropic, enabling their roles in orchestrating complex biological processes [25,26,27,28]. While our focus is on transmembrane GPCRs, emerging evidence strongly indicates that GPCR signaling also occurs from intracellular compartments [29]. GPCRs are localized in the membranes of endosomes, the nucleus, the mitochondria, and other organelles, where they are closely linked to metabolic functions [29,30,31]. For further details on intracellular GPCRs in metabolic regulation, please refer to the cited studies.

GPCRs play a significant role in human pathophysiology and responsiveness to pharmacological interventions, making them among the most thoroughly investigated drug targets [32,33,34]. Remarkably, 34% of drugs currently available in the market achieve their therapeutic effects by modulating GPCR activity, many of which target common diseases [33,35].

GPCRs are especially important in metabolism, where dysregulated GPCR signaling has been closely related to obesity and related metabolic diseases [36,37,38,39,40]. For instance, G-protein-coupled receptors 40 (GPR40) and 120 (GPR120), activated by long-chain fatty acids, play pivotal roles in pancreatic insulin secretion and inflammatory responses, with GPR120 also regulating appetite control, adiposity, and food preference [41,42,43,44,45,46]. Genetic studies using mouse models suggest that GPR120 dysfunction contributes to diet-induced obesity and related metabolic disorders [47,48]. Glucagon-like peptide-1 receptor (GLP-1R) influences both the synthesis and secretion of insulin, contributing to the maintenance of euglycemia [49,50,51]. β-adrenergic receptor signaling promotes lipid catabolism in adipose tissue and thermogenesis, highlighting their involvement in energy expenditure [51,52,53,54,55]. Mutations in these receptors can lead to the onset and progression of obesity and type 2 diabetes [55,56,57]. As a result, these receptors present potential targets for therapeutic interventions for both conditions.

**Figure 1:**
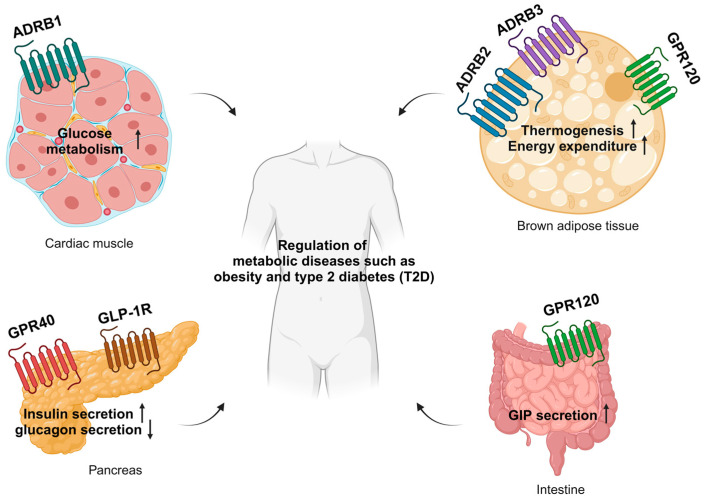
Overview of key GPCRs involved in the regulation of metabolic functions and diseases. ADRB1 in cardiac muscle regulates glucose metabolism. ADRB2, ADRB3, and GPR120 regulate brown adipose function. GPR40 and GLP-1R in the pancreas regulate insulin homeostasis; GPR120 in the intestine activates GIP secretion. Metabolic diseases can be treated by pharmacologically targeting these GPCRs. GIP, gastric inhibitory polypeptide. The figure was created using BioRender (http://biorender.com/accessed on 21 January 2025).

In this review, we focus on four key metabolic GPCRs—GPR40, GPR120, GLP-1R, and β-adrenergic receptors (ADRB1, ADRB2, and ADRB3)—with respect to their roles in metabolic physiology and significance in obesity and related metabolic diseases (Figure 1).

## 2. GPR40

### 2.1. GPR40 Signaling in Metabolic Physiology

**Figure 2:**
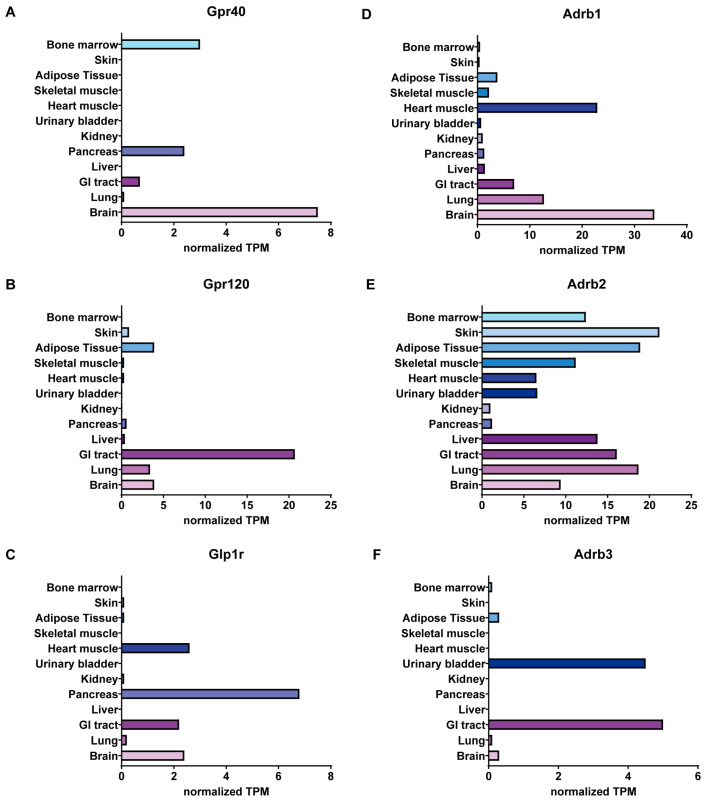
mRNA expression of GPCRs in metabolic organs; (**A**–**F**) Normalized TPM values of Gpr40, Gpr120, Glp1r, Adrb1, Adrb2, and Adrb3 expressed in organs that can contribute significantly to maintaining metabolic homeostasis. Data was processed from RNA expression data of Human Protein Atlas (v24.proteinatlas.org, accessed on 13 January 2025) [61].

GPR40, or free fatty acid receptor 1 (FFAR1), is predominantly expressed in the brain, pancreatic β-cells, and intestinal enteroendocrine cells (L, K, and I cells) (Figure 2) [58,59]. GPR40 was first deorphanized in pancreatic β-cells and identified as an insulin secretion activator (Figure 1) [41]. When stimulated by its endogenous agonists, free fatty acids, or synthetic agonists, GPR40 activates the Gαq, which in turn activates phospholipase C (PLC), converting plasma membrane phosphatidylinositol-4,5-bisphosphate (PIP2) into inositol-1,4,5-trisphosphate (IP3) and diacylglycerol (DAG). IP3 then moves into the endoplasmic reticulum (ER), causing the release of stored calcium from the ER, which raises cytoplasmic calcium levels. That elevation of calcium concentration leads to glucose-stimulated insulin secretion [41,59]. DAG activates protein kinase C (PKC), leading to enhanced glucose oxidation coupled with mitochondrial respiration. This activation supports the glycerolipid/free fatty acid cycle (GL/FFA), which in turn stimulates insulin secretion [60]. Activation of GPR40 also stimulates the secretion of glucagon and other incretins, which facilitate insulin release by β-cells. GPR40 agonism in enteroendocrine cells also enhances incretin secretion. Due to these functions in insulin homeostasis, GPR40 has emerged as a promising therapeutic target for antidiabetic agents.

### 2.2. Pharmacology of GPR40

Fatty acids, an endogenous ligand of GPR40, are key nutrients after meal ingestion. Once absorbed in the gut, fatty acids not only provide an energy source but also medium- and long-chain free fatty acids act as agonists for GPR40 [62]. Linolenic acid (C18:2) is a native 18-carbon fatty acid ligand and is utilized as a reference ligand for GPR40 and GPR12 (Table 1). While α-linolenic has a high affinity for both GPR40 and 120, oleic acid (C18:1) has an affinity for GPR40 but not for GPR120 [63]. The GPR40 selective and partial agonist Fasiglifam (TAK-875) significantly lowered glucose levels in type 2 diabetes patients, minimizing the risk of hypoglycemia and weight gain via glucose-stimulated insulin release [64]. Despite the proven efficacy in human and phase III clinical studies, this agonist was withdrawn due to off-target and liver toxicity [65,66]. To improve the toxicity, CPL207280, a para-alkoxyphenylpropionic acid derivative, was developed from TAK-875 through structural modifications focused on lowering molecular weight and lipophilicity. These modifications result in CPL207280 demonstrating threefold higher potency (EC_50_ = 80 nM) compared to TAK-875 (EC50 = 270 nM) [67]. Additionally, CPL207280 significantly enhanced glucose-stimulated insulin release and improved glucose tolerance in murine pancreatic MIN6 cell lines, primary β-cells, healthy Wistar Han rats, and diabetic rat models [55,67,68]. In the first-in-human clinical study (phase 1 and phase 2), CPL207280 was found to be liver-safe and well tolerated by healthy volunteers, showing a low risk of liver toxicity [69].

Following the discovery of the partial agonist AMG-837, which enhanced glucose-stimulated insulin secretion and lowered post-prandial glucose levels in a preclinical test but failed in the clinical test due to toxicity [70]. Amgen focused on the optimization of AMG-837 to identify a series of full agonists such as AM-1638 or AM-6226 [71,72]. Indeed, these full agonists showed excellent preclinical results and entered the clinical trials showing better agonistic activity in diabetes treatment compared to the partial agonist AMG-837 in clinical trials [73]. In particular, AM-6226 demonstrated a greater effect on glucose lowering in cynomolgus monkeys, suggesting that it maintains glycemic control in non-human primates.

On the other hand, the GPR40 full agonist SCO267, developed by SCOHIA PHARMA, displayed an ability to stimulate insulin secretion, promote GLP-1 release, and improve glucose tolerance [74,75]. Currently, it is under investigation in a Phase II clinical trial.

### 2.3. Downstream G-Protein Pathways of GPR40

GPR40 signaling is primarily mediated through the Gαq/11 pathway, which is activated upon ligand binding [59]. This activation triggers intracellular calcium mobilization, facilitating downstream signaling that enhances glucose-dependent insulin secretion in pancreatic β-cells [76]. Notably, pharmacological blockade of Gαq signaling significantly reduces insulin secretion, confirming its essential role in the GPR40-mediated insulinotropic effect. However, Gαs activation of GPR40 has also been observed, particularly in enteroendocrine cells. Gαs activation stimulates adenylyl cyclase, leading to increased cyclic adenosine monophosphate (cAMP) levels, which in turn promotes glucagon-like peptide-1 (GLP-1) release from enteroendocrine cells [77]. Recent studies suggest that GPR40 signaling may exhibit pathway-specific or biased activation depending on the ligand [77,78]. For instance, TAK-875, a partial agonist, preferentially couples to Gαq-mediated calcium signaling, whereas full agonists like AM-1638 and SCO267 enable dual coupling to both Gαq/Ca^2+^ and Gαs/cAMP pathways.

This signaling bias is further supported by structural insights. The crystal structure of GPR40 bound to the TAK-875 revealed that this synthetic agonist interacts with distinct allosteric sites [79]. Furthermore, a second structurally distinct allosteric site has been identified, which is generally targeted by full agonists such as AMG-1638, and computational modeling suggests that the endogenous free fatty acid, γ-linolenic acid, can also occupy this site [80]. These findings provide structural evidence that agonist binding at different allosteric sites on the same receptor can modulate the receptor’s affinity for various downstream G proteins, resulting in biased G-protein signaling.

## 3. GPR120

### 3.1. GPR120 Signaling in Metabolic Physiology

GPR120, also known as free fatty acid receptor 4 (FFAR4), is expressed in various cell types, including enteroendocrine cells, as well as pancreatic α- and δ-cells, but is notably absent from β-cells [81]. GPR120 is also found in macrophages, adipocytes, taste buds, and the gastrointestinal tract (Figure 2) [82]. The widespread distribution of GPR120 throughout the body suggests its involvement in regulating many biological processes, including anti-inflammatory, neuroprotective, antiproliferative, and antidiabetic functions [63,82].

In the context of adipose tissues, GPR120 is expressed in both white adipose tissue (WAT) and brown adipose tissue (BAT), with its expression in BAT further increasing in response to cold exposure (Figure 1) [83,84]. The activation of GPR120 has been shown to stimulate non-shivering thermogenesis, as evidenced by the increased expression of thermogenic markers such as uncoupling protein 1 (UCP1) in BAT. Furthermore, GPR120-deficient mice exhibit a reduced ability to induce browning of subcutaneous white adipose tissue (WAT) when exposed to cold exposure [83]. GPR120 stimulation induces WAT browning by stimulating the secretion of fibroblast growth factor 21 (FGF21) [84]. Detailed physiology regarding thermogenic adipose tissues above will be further discussed in the ADRB section.

A significant body of evidence supports GPR120’s role in preventing diet-induced obesity through mechanisms involving the regulation of adipogenesis and adipocyte lipid handling. Studies using GPR120 KO mice fed with a high-fat diet (HFD) demonstrated that these mice accumulate significantly more fat mass compared to the wild-type group [48]. Additionally, activation of GPR120 has been linked to improved insulin homeostasis. In obese mice, the administration of a GPR120-selective agonist has shown promising effects in enhancing glucose tolerance, improving insulin sensitivity, reducing hyperinsulinemia, and alleviating hepatic steatosis, all likely due to the increased insulin secretion [85,86].

In the gastrointestinal tract, GPR120 is expressed in K, L, and I cells, where its activation induces the secretion of glucose-dependent insulinotropic polypeptide (GIP) and cholecystokinin (CCK), both of which play critical roles in regulating digestion and glucose metabolism (Figure 1) [87,88,89]. Within pancreatic islets, GPR120 is primarily expressed in somatostatin (SS)-secreting δ-cells and pancreatic polypeptide (PP)-secreting γ-cells, leading to the inhibition of somatostatin secretion [90]. In contrast, the Gαq/11 protein in γ-cells enhances calcium signaling, which in turn stimulates the secretion of pancreatic polypeptide (PP) [91].

These findings collectively highlight the critical role of GPR120 in metabolic regulation, energy homeostasis, and endocrine signaling, further establishing its potential as a therapeutic target for conditions such as obesity, diabetes, and metabolic syndrome.

### 3.2. Pharmacology of GPR120

Medium- and long-chain fatty acids serve as agonists for both GPR120 and GPR40, while short-chain fatty acids activate other members of the free fatty acid receptor family, such as GPR41 and GPR43 [92]. Endogenous agonists for GPR120 include 9-hydroxystearic acid (9-HSA), linoleic acid, oleic acid, and omega-3 (ω-3) eicosapentaenoic acid (EPA), a major component of fish oil (Table 1).

The development of synthetic ligands for GPR120 was driven by amino acid sequence similarity between GRP120 and GPR40. This overlap led to the discovery that many established GPR40 agonists also activate GPR120. One such agonist, GW-9508, served as the starting point for generating a series of derivatives targeting GPR120. Among these, TUG-891 stands out as a selective GPR120 agonist, demonstrating a 1000-fold greater potency for GPR120 compared to GPR40 [93].

### 3.3. Downstream G-Protein Pathways of GPR120

Since both GPR40 and GPR120 belong to the fatty acid receptor family, their activation and signaling by free fatty acids are molecularly similar [94]. GPR120 mediates insulin sensitization, incretin hormone secretion, and anti-inflammatory response through its interaction with various downstream effectors, including G proteins (Gαs, Gαi, and Gαq) and β-arrestins [47]. Two splicing variants of GPR120 exist: a short isoform (GPR120S) and a long isoform (GPR120L). GPR120L contains an additional 16 amino acids (residues 231-247) within the third intracellular loop, which is absent in GPR120S. This region is essential for signaling selectivity, as GPR120S couples Gαs, Gαi, Gαq, and β-arrestins, while GPR120L loses its ability to couple with Gαq and Gα [95].

Ligand-dependent pathway activation or bias signaling of GPR120 has been demonstrated through assays such as a BRET (Bioluminescence resonance energy transfer) and Gα-Gγ dissociation assay, which reveal distinct agonist-specific signaling profiles. For example, 9-HSA, EPA, and TUG-891 exhibit comparable effects on Gαi signaling. However, TUG-891 shows enhanced Gαq activity relative to 9-HSA and EPA, indicating that TUG-891 functions as a Gαq-biased ligand [95]. Conversely, EPA demonstrates greater efficacy in Gαs signaling, whereas TUG-891 or 9-HSA exhibit minimal activity, suggesting that EPA acts as a Gαs biased ligand [95]. Supporting this phenomenon, recent in vivo studies have shown that linoleic acid demonstrates greater potency than oleic acid in enhancing systemic insulin secretion, a process that relies on the β-arrestin-2 function [96].

## 4. GLP-1R

### 4.1. GLP-1R Signaling in Metabolic Physiology

Glucagon-like peptide-1 receptor (GLP-1R) is highly expressed in islet β-cells and minimally in α- and δ-cells, but can also be found in several organs, like lungs, kidneys, stomach, heart, intestine, adipose tissue, and multiple regions of the central nervous system (CNS) such as the hypothalamus and brain stem (Figure 2) [97]. GLP-1 is a peptide hormone composed of 30 or 31 amino acids and is secreted by enteroendocrine L cells, pancreatic islet α-cells, and neurons in the nucleus of the solitary tract. This distribution underscores GLP-1’s diverse physiological roles in glucose metabolism, insulin secretion, and neuroendocrine regulation. Original GLP-1 (1–37) undergoes amidation and proteolytic cleavage, resulting in the formation of two biologically active and equally potent truncation variants: GLP-1 (7–36) and GLP-1 (7–37).

So far, the best-understood role of GLP-1R signaling in metabolic physiology is its function in the pancreas and brain [98,99]. In the pancreas, activation of GLP-1R promotes insulin secretion in a glucose-dependent manner, supporting blood sugar control, which is supported by the fact that a GLP-1R knockout disrupted pancreatic function, leading to imbalances in hormone regulation and impairing glucose homeostasis [100,101]. This effect is mediated by the stimulation of adenylyl cyclase, which increases cAMP levels and triggers pancreatic insulin release. In the brain, GLP-1R is found in areas such as the hypothalamus and brainstem, where its activation regulates appetite and promotes satiety. By influencing neuronal activity in these regions, GLP-1R signaling reduces food consumption and helps maintain energy balance. These diverse functions make GLP-1R a key therapeutic target for managing diabetes and obesity.

### 4.2. Pharmacology of GLP-1R

GLP-1 plays a critical role in glucose homeostasis following food consumption by binding to the GLP-1 receptor. However, the direct use of GLP-1 as a clinical therapeutic agent is limited by its short half-life of approximately 2 min, primarily caused by rapid degradation by the enzyme dipeptidyl peptidase-IV. To overcome this limitation, GLP-1 receptor agonists (GLP-1RAs) have been developed, which mimic the action of endogenous GLP-1. These agonists activate GLP-1R, enhancing insulin secretion, inhibiting glucagon release, delaying gastric emptying, and suppressing appetite through central mechanisms, making them valuable therapeutic agents for metabolic disorder [102].

The development of GLP-1RAs has been driven by the success of peptide-based drug exenatide, the first FDA-approved GLP-1 analog with 50% amino acid homology to GLP-1 and an extended half-life of up to 2.4 h [103,104,105]. This milestone has paved the way for other FDA-approved GLP-1 analogs, such as liraglutide and semaglutide, with half-lives of 11 to 13 h and 144 to 168 h, respectively [106,107] (Table 1), highlighting the broad therapeutic potential of GLP-1RAs in treating systemic diseases, including type 2 diabetes and obesity.

Currently, most GLP-1RAs are large protein- or peptide-based molecules that are typically administered via injection. In contrast, small molecules GLP-1RAs, which are chemically synthesized and have smaller molecular size, are emerging as promising alternatives that enable oral administration. The development of small molecules GLP-1RAs aims to address several limitations associated with peptide-based drugs, such as the delivery only available through injection.

Despite their potential, research on small molecule GLP-1RAs remains largely in the laboratory and clinical trial stage. One notable example of a small molecule GLP-1RA is Orforglipron (LY3502970/OWL833), an oral agent developed by the Eli Lilly and Chia Tai Tianqing Pharmaceuticals Group [108,109]. In a phase II study targeting patients with type 2 diabetes, Orforglipron demonstrated a significant reduction in glycated hemoglobin (HbA1c) and weight, meeting primary and secondary endpoints [110]. Currently, Eli Lilly has initiated a phase III development program to further investigate the efficacy and safety of Orforglipron in treating obesity, overweight, and type 2 diabetes [110]. Recent studies have also other small molecules GLP-1RAs such as Danuglipron, or GSBR-1290 are under evaluation through early-phase clinical trials [111,112].

### 4.3. Downstream G-Protein Pathways of GLP-1R

Upon activation by the agonists, GLP-1R couples to various Gα proteins. The predominant signaling pathway regulating GLP-1R activity is believed to involve coupling with Gαs, which activates adenylyl cyclase and increases the production of cAMP [113]. Protein kinase A (PKA), a tetrameric enzyme consisting of two catalytic subunits [114], is known to be the primary target for cAMP. PKA functions as the main effector mechanism for G-protein-coupled receptors that are connected to adenylate cyclase [115]. The cAMP/PKA signaling enhances insulin secretion and glucose sensitivity in pancreatic β-cells.

In addition to coupling with the Gαs/cAMP/PKA pathway, GLP-1R can interact with other G protein subtypes, including Gαi, Gαq, Gαo, and Gα11 [116]. Furthermore, downstream signaling in β-cells is mediated by β-arrestins, specifically β-arrestin-1 and β-arrestin-2. β-arrestin-1 promotes the phosphorylation of cAMP response element binding protein (CREB) and extracellular signal-regulated kinase (ERK1/2) [116], which in turn leads to the phosphorylation of Bad (Bcl-xL/Bcl-2-associated death promoter homolog), thereby preventing β-cells apoptosis [117]. Additionally, β-arrestin-2 plays a crucial role in regulating insulin secretion. In mouse models, deletion of β-arrestin-2 results in impaired insulin release, indicating its essential function in β-cell signaling [118].

## 5. ADRB/β-AR

Adrenoreceptor beta (ADRB), known as a β-adrenergic receptor (β-AR), consists of three isoforms: ADRB1/β1-AR, ADRB2/β2-AR, and ADRB3/β3-AR [119]. These receptors are classically involved in whole-body catabolism by increasing glycogen breakdown and enhancing metabolic activity during immediate stress responses (fight-or-flight). All three isoforms share a common endogenous ligand—neurohormonal catecholamines such as norepinephrine—released from sympathetic nerve terminals and/or the adrenal glands. Biochemical studies suggest they may bind to each β-AR isoform with different affinities, though the exact degree of binding specificity and underlying mechanisms require further investigations [120,121].

Each ADRB isoform is distinctly expressed in metabolic tissues (Figure 2). For example, ADRB1/β1-AR is predominantly expressed in the heart, particularly in cardiac muscle and brain, with modest expression in the lung, GI tract, and adipose tissues (Figure 2). Among the three β-AR isoforms, ADRB2/β2-AR is the most ubiquitously expressed throughout the tissues, whereas ADRB3/β3-AR has a more selective expression pattern, primarily found in the bladder, GI tract, and adipose tissues (Figure 2).

Upon activation, β-ARs couple with the Gαs subunit, which subsequently activates adenylyl cyclase, leading to the conversion of ATP to cAMP. The increased cAMP levels activate PKA, a critical effector that regulates multiple downstream signaling pathways [22,122]. Under certain conditions, β-ARs can also interact with Gαi proteins, which antagonize the Gαs pathway by inhibiting adenylyl cyclase, thereby reducing cAMP levels and inactivating PKA. For example, PKA-mediated β2-AR phosphorylation can switch its G-protein coupling from Gαs to Gαi, leading to adenylyl cyclase inhibition, a reduction in cAMP levels, and subsequent PKA inactivation [123]. This dual signaling mechanism allows fine-tuned regulation of β-AR pathways in different tissues.

Beyond direct G protein signaling, beta-adrenergic receptor kinase (βARK1 or GRK2) is an important regulator of β-AR activity. It phosphorylates activated β-adrenergic receptors (β-ARs) to promote their desensitization and internalization [124]. GRK2-mediated phosphorylation of β1-AR and β2-AR leads to β-arrestin recruitment, which not only deactivates G protein signaling but also initiates distinct β-arrestin-dependent pathways that activate cardiac contractility [125]. Dysregulated GRK2 expression has been implicated in cardiovascular diseases, particularly heart failure, where its upregulation contributes to impaired β-adrenergic signaling and reduced cardiac responsiveness to catecholamines [126]. Genetic knockout studies in mice have demonstrated that GRK2 is essential for early heart development, as its loss results in embryonic lethality due to severe cardiac defects [124]. GRK2 also modulates other GPCRs and receptor tyrosine kinases (RTKs), such as the epidermal growth factor receptor (EGFR), influencing cardiac hypertrophy and survival pathways [127]. This regulatory role further supports the notion that ADRB1-mediated EGFR transactivation occurs through the formation of direct receptor–receptor interaction [128]. Pharmacological inhibition of GRK2 has emerged as a promising therapeutic strategy for heart failure, with inhibitors showing potential in restoring β-AR function and improving cardiac output [125].

### 5.1. ADRB1/β1-AR Signaling in Metabolic Physiology

In the heart, β1-AR constitute approximately 80% of total cardiac β-Adrenergic receptors, making them the dominant β-AR subtype in cardiac tissue [129]. In ventricular myocytes, β1-AR stimulation triggers the Gαs-adenylyl cyclase-cAMP-PKA signaling cascade, which phosphorylates diverse intracellular cardiac proteins, including phospholamben, cardiac troponin I, L-type calcium channels, and cardiac myosin-binding protein C [130]. This phosphorylation, in general, enhances cardiac contractility and heart rate, which is important for adaptive responses to stress and exercise [131,132,133]. Recent studies have also linked β1-AR signaling to insulin-mediated glucose uptake in cardiomyocytes (Figure 1). Activation of β1-AR promotes AKT phosphorylation at Threonine 308, facilitating glucose transport into cardiac cells through GLUT4’s trafficking to the plasma membrane [134].

All three β-AR isoforms are expressed in both white adipose tissues (WAT), including subcutaneous and visceral WAT, and brown adipose tissue (BAT). However, their expression levels vary depending on the specific adipose depot, species, the differentiation state of adipocytes [52]. In response to cold exposure, β1-AR signaling in adipose tissue cooperates with β3-AR to trigger lipolysis in mature adipocytes, a lipid breakdown process that generates free fatty acids (FFAs) as an energy source for other tissues. Indeed, a recent study showed both β1- and β3-AR mRNA are increased upon cold exposure, like from thermoneutral condition (30 °C) to cold temperature (4–6 °C) [135]. That said, β3-AR, rather than β1-AR, serves as the primary receptor responsible for cold-induced lipolysis, a topic that will be discussed in greater detail in the β3-AR section.

Conversely, β1-AR plays a more critical role in adipocyte precursor cells. Early in vitro studies suggest that norepinephrine triggers cAMP production and brown preadipocyte proliferation via β1-AR signaling, which aligns with the dominant expression of ADRB1 in adipocyte stem and progenitor cells (ASPCs), whereas ADRB3 is abundantly expressed in mature adipocytes [136]. Furthermore, β1-AR signaling has been shown to stimulate BAT adipogenesis following prolonged cold acclimation [136]. Genetic studies using mouse models demonstrated that global β1-AR deficiency impairs ASPCs proliferation and prevents the recruitment of new adipocytes following norepinephrine stimulation, indicating that norepinephrine-regulated β1-AR signaling is indispensable for de novo brown adipogenesis [137]. However, recent studies using preadipocyte-specific β1-AR KO mouse models suggest that cold-induced brown adipocyte progenitor proliferation does not require direct β1-AR signaling, emphasizing the need for further studies to determine which cell types mediate this process [138].

### 5.2. Pharmacology of ADRB1/β1-AR

Norepinephrine and epinephrine are the endogenous agonists of ADRB1 as well as all subtypes in an adrenergic receptor family (Table 1). Isoproterenol also stimulates ADRB1 as a synthetic ligand and has efficacy comparable to endogenous agonists across all beta-adrenergic receptor subtypes, without selectivity for any specific subtypes [139]. Therefore, it has been utilized for β-ARs’ pan-agonists. But it also binds to α-adrenergic receptors, showing bias activation for α1A adrenergic receptor [140].

As previously mentioned, ADRB1, along with other β-adrenergic receptor subtypes such as ADRB2 and ADRB3, are expressed in cardiac muscle. Therefore, β-blockers, which act as antagonists to β-Ars, are commonly used for the treatment of many cardiovascular diseases, including heart failure, acute and chronic ischemic heart disease, tachyarrhythmias, and hypertension. These drugs primarily antagonize the ADRB1. Propranolol, a nonselective β-AR blocker, is widely used for the treatment of hypertension. CGP20712A, a selective ADRB1 antagonist, demonstrated an IC50 of 1.6 nM for ADRB1 and an IC50 of 776 nM for ADRB2 [141] (Table 1). Similarly, Atenol is also a selective ADRB1 antagonist, but it has a weaker IC50 of 219nM for ADRB1 [141].

### 5.3. Downstream G-Protein Pathways of ADRB1/β1-AR

Upon agonist binding, ADRB1 predominantly couples with the Gαs protein, activating adenylyl cyclase and increasing cAMP levels [142]. In addition to this classic pathway, other G protein subtype interactions have also been identified, including coupling with Gαz and Gα12, which broaden ADRB1’s signaling repertoire. These pathways are implicated in calcium mobilization and Rho pathway activation, respectively, contributing to diverse physiological outcomes [143]. Beyond G protein-mediated signaling, in ADRB1, β-arrestin mediates activation of ERK1/2 and other downstream pathways, which are critical for cardioprotection and cellular remodeling [144]. Recent studies report the importance of ADRB1-mediated EGFR transactivation, facilitated by β-arrestin, in promoting cardioprotective signaling and modulating ERK1/2 activity. While G protein signaling primarily enhances heart rate and activates canonical cardiac contractility via cAMP production, β-arrestin-dependent pathways contribute to receptor internalization, cytosolic ERK retention, and cardioprotective activity against chronic adrenergic stimulation [145,146]. However, β-arrestin-dependent signaling is less prominent in ADRB1 compared to ADRB2 [147].

### 5.4. ADRB2/β2-AR Signaling in Metabolic Physiology

Acute β2-AR stimulation enhances insulin-induced glucose uptake by facilitating GLUT4 translocation to the plasma membrane and potentiates and enhances cardiac contractility through increased cAMP-dependent PKA activation [134]. Chronic β2-AR activation, however, results in persistent AKT signaling, which paradoxically leads to downregulation of GLUT4 expression and impairs its membrane translocation, potentially contributing to insulin resistance in metabolic tissues [148].

Catecholamines-stimulated glycogenolysis occurs primarily through β2-ARs under normal physiological conditions, particularly in the liver. Ectopic hepatic β2-AR signaling can modulate glucose metabolism by altering the expression of key gluconeogenic and glycolytic enzymes, including phosphoenolpyruvate carboxykinase (PEPCK), glucose-6-phosphatase (G6PC), and pyruvate kinase (PK), thereby impacting glucose production and utilization [149].

The functional significance of β2-AR in adipose tissue biology remained largely undetermined until somewhat overlooked until recent studies highlighted its importance. One possible reason for this oversight is its low expression in mature adipocytes in murine models. Instead, β2-AR is predominantly expressed in the vascular cells, where its activation promotes increased blood flow to BAT. In contrast, human brown adipocytes express β2-AR at significantly higher levels, and β2-stimulation is essential for human BAT lipid catabolism and thermogenesis (Figure 1) [150]. These findings underscore the critical role of β2-signaling in adipocyte biology, particularly in regulating energy homeostasis and thermogenic activity in humans.

### 5.5. Pharmacology of ADRB2/β2-AR

ADRB2 also can be stimulated by norepinephrine and isoproterenol. Formoterol is a long-acting beta-2 agonist (LABA) used for asthma and chronic obstructive pulmonary disease (COPD) (Table 1) [151]. It has a higher affinity for ADRB2 and shows ADRB2 selectivity towards ADRB1 and ADRB3 [141]. Through cAMP accumulation assay in U937 promonocytes, the Emax and EC50 values of various agonists for B2-AR were determined: isoproterenol (pEC50 = 8.58 ± 0.10), epinephrine (pEC50 = 7.70 ± 0.08), norepinephrine (pEC50 = 5.69 ± 0.07), salbutamol (pEC50 = 6.95 ± 0.07), Fenoterol (pEC50 = 8.23 ± 0.09), and Formoterol (pEC50 = 9.61 ± 0.12) [152].

### 5.6. Downstream G-Protein Pathways of ADRB2/β2-AR

While ADRB2’s G protein and β-arrestin signaling are well characterized in the cardiac system—enhancing heart rate and contractility through Gαs and cAMP while providing cardioprotection through Gαi and β-arrestin-mediated pathways [153,154,155]—its role in other tissues is relatively less understood. ADRB2 G protein signaling stimulates cAMP production, driving adipose tissue lipolysis, pancreatic glucose homeostasis, and peripheral tissue glucose uptake to regulate energy balance [156,157,158].

Beyond G protein signaling, β-arrestin activates alternative pathways such as MAPK and AKT, promoting glucose uptake and energy expenditure [159,160]. Dysregulated ADRB2 signaling is implicated in obesity and diabetes, and although β-arrestin’s metabolic roles are less studied, they hold potential for therapeutic strategies targeting metabolic disorders by improving energy metabolism and flexibility.

### 5.7. ADRB3/β3-AR Signaling in Metabolic Physiology

One of the contexts in which the physiological roles of β3-AR have been extensively studied is adipose tissue lipid catabolism and thermogenesis [139,161]. For example, lipolysis, induced during cold exposure, exercise, and fasting, stimulated by catecholamines and glucagon, occurs via a Gαs-cAMP-PKA-mediated mechanism, leading to the activation of key lipases: adipose triglyceride lipase (ATGL) and hormone-sensitive lipase (HSL). This activation enhances the breakdown of triglycerides stored in lipid droplets into fatty acids and glycerol [162]. The free fatty acids are transported into the bloodstream to be used as energy by other tissues, while glycerol is sent to the liver for gluconeogenesis [162].

In response to β3-AR signaling, thermogenic adipocytes—brown adipocytes and beige adipocytes (brown-like adipocytes located in subcutaneous adipose tissue)—increase the utilization of free fatty acids from circulation, fatty acids produced intrinsically by the cell, or glucose as energy sources [52,162]. Using these fuels, these adipocytes expend energy in the form of heat by combining conventional fuel oxidation processes (glycolysis, β-oxidation, and the TCA cycle) with a distinct mitochondrial uncoupling mechanism mediated by uncoupling protein 1 (UCP1), a process known as non-shivering thermogenesis [52]. UCP1, embedded in the mitochondrial inner membrane, facilitates the leakage of protons (H^+^) along the proton gradient across the mitochondrial inner membrane, converting this gradient into heat. This mechanism effectively uncouples aerobic ATP generation [52].

### 5.8. Pharmacology of ADRB3/β3-AR

Mirabegron has been used to stimulate human β3-ARs and approved for the treatment of individuals with overactive bladders (Table 1) [163]. Furthermore, it has been reported that Mirabegron can increase human whole-body expenditure by stimulating brown adipose tissue (BAT) recently [150]. CL-316,243 is also a selective ADRB3 agonist and has been used to mimic the cold-induced thermogenesis of BAT in mice (Figure 1) [164]. The global ADRB3 KO mice eliminate blood glucose clearance by CL-316,243, and this response is restored when ADRB3 are reintroduced in WAT and/or BAT [165].

### 5.9. Downstream G-Protein Pathways of ADRB3/β3-AR

The adipocyte β3-AR-Gαs-cAMP-PKA pathway transcriptionally regulates non-shivering thermogenesis by activating the downstream transcription factors, including the cAMP-responsive element binding protein (CREB), which targets genes involved in UCP1 expression and mitochondrial metabolism [166,167,168]. Interestingly, β-arrestin signaling, which acts as a negative modulator of the G protein pathway, also plays a significant regulatory role for this receptor. For example, studies in mice with adipocyte-specific deletion of β-arrestin-2 have shown enhanced ADRB3 signaling, leading to protection against weight gain and overall metabolic improvement. These findings demonstrate that the β-arrestin, which interacts with ADRB3, acts as a negative regulator of β3-AR signaling in vivo [169,170].

## 6. Limitation and Future Perspectives in GPCR-Targeted Therapeutics for Metabolic Diseases

As the intricate role of GPCRs in metabolic regulation continues to unfold, the potential for targeted therapies aimed at obesity, type 2 diabetes, and related metabolic disorders grows increasingly promising. The emerging pharmacological landscape, focusing on receptors such as GPR40, GPR120, GLP-1R, and adrenergic receptors (ADRB1, ADRB2, ADRB3), underscores the need for continued innovation in this field. Despite notable advances, critical challenges and opportunities lie ahead that will shape the future of GPCR-targeting therapeutics.

Despite the promising role of GPR120 and GPR40 in metabolic regulation, several challenges complicate the development of effective therapeutics targeting these receptors. One major limitation in GPR40-based drug discovery is the risk of off-target effects and hepatotoxicity, as exemplified by the discontinuation of fasiglifam (TAK-875) during phase III clinical trials due to liver toxicity. This underscores the need for structural optimization and the development of ligands with improved safety profiles.

For GPR120, the complexity of biased signaling presents both an opportunity and a challenge. While biased agonists can selectively activate beneficial pathways, the pleiotropic nature of GPR120 signaling increases the likelihood of unpredictable pharmacological responses. Additionally, the expression of GPR120 in multiple tissues, including adipose, pancreatic, and immune cells, complicates tissue-specific targeting, raising concerns about unintended side effects and limiting the therapeutic window.

The concept of biased signaling, whereby ligands preferentially activate specific GPCR pathways, represents a paradigm shift in drug development. This approach allows for the fine-tuning of receptor activity to enhance therapeutic efficacy while minimizing side effects. In the case of GPR40 and GPR120, the discovery of full agonists such as SCO267, which engage distinct allosteric sites, exemplifies the potential for pathway-specific modulation. Future research aimed at elucidating the structural basis of allosteric modulation and biased signaling will be critical for optimizing receptor targeting.

For the GLP-1 receptor, agonists (GLP-1RAs) such as semaglutide and liraglutide have demonstrated remarkable efficacy in managing obesity and type 2 diabetes. However, issues such as injection-based delivery and patient adherence present barriers to widespread adoption. The ongoing development of small molecule GLP-1RAs, exemplified by orforglipron and danuglipron, represents a transformative shift. Although these orally available agents need to be tested in clinical trials, they aim to overcome the limitations associated with peptide-based therapies, offering greater convenience and broader patient accessibility.

Similarly, while adrenergic agonists like mirabegron, an FDA-approved drug for overreactive bladder disease, have proven effective in stimulating thermogenesis and promoting weight loss, their cardiovascular side effects necessitate the exploration of more selective, tissue-specific agents. Designing positive allosteric modulators that potentiate β3-adrenergic receptors or biased ligands that selectively activate beneficial signaling pathways of this receptor without triggering adverse off-target effects holds significant therapeutic promise.

While current efforts focus on a limited subset of metabolic GPCRs, expanding the repertoire of targetable receptors remains a key objective. Receptors such as M3 muscarinic acetylcholine receptor (M3R) [171,172] melanocortin-4 receptor (MC4R) [173], ghrelin receptor (GHSR) [174], glucose-dependent insulinotropic polypeptide receptor (GIPR) [175] and taste receptors [176] present compelling opportunities for therapeutic intervention. Emerging evidence suggests that combinatorial targeting of multiple GPCRs may yield synergistic effects, providing a more comprehensive approach to addressing the multifaceted nature of metabolic diseases.

Moreover, the integration of designer receptors exclusively activated by designer drugs (DREADDs) offers a novel avenue for selectively modulating GPCR activity [177]. This technology, currently in experimental stages, has the potential to overcome limitations related to non-specific pathway or adverse systemic effects, providing unprecedented control over GPCR signaling in specific tissues.

## 7. Conclusions

The future of GPCR-targeted therapeutics in metabolic disease management presents exciting opportunities for innovation and discovery. By addressing current limitations, embracing novel technologies, and expanding the repertoire of targetable receptors, the field is poised to unlock new frontiers in precision medicine for the treatment of obesity, type 2 diabetes, and related metabolic disorders.

## Figures and Tables

**Table 1:** Properties and chemical structure of key ligands of GPR40, GPR120, GLP-1R, ADRB1, ADRB2 and ADRB3.

Receptor	Ligand	Property	Chemical Formula
GPR40 (FFA1)	linoleic acid	endogenous agonist	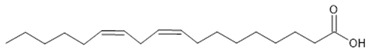
	oleic acid	endogenous agonist	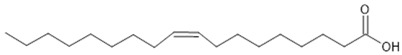
	fasiglifam (TAK-875)	β-arrestin-biased agonist, partial agonist of the Gαq signaling	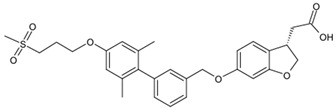
	CPL207280	agonist	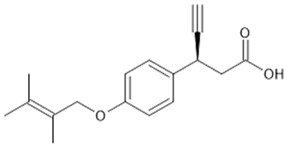
	AMG-837	partial agonist	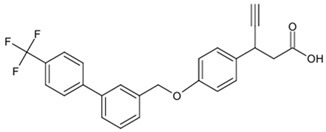
	AMG-1638	full agonist	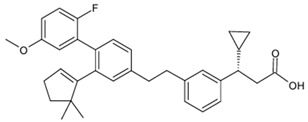
	AMG-6226	partial agonist	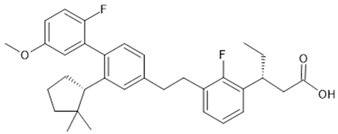
	SCO267	full agonist	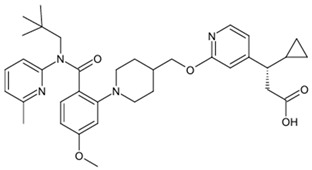
GPR120 (FFA4)	TUG-891	selective GPR120 agonist	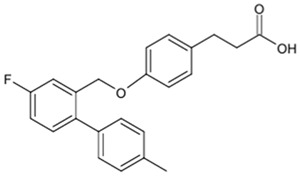
	9-HSA	endogenous agonist	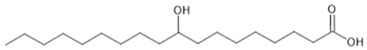
	EPA	endogenous agonist	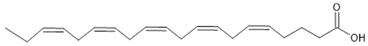
	linoleic acid	endogenous agonist	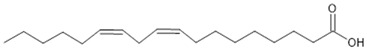
	oleic acid	endogenous agonist	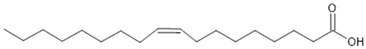
GLP-1R	liraglutide	lipopeptide	His-Ala-Glu-Gly-Thr-Phe-Thr-Ser-Asp-Val-Ser-Ser-Tyr-Leu-Glu-Gly-Gln-Ala-Ala-Lys-Glu-Phe-Ile-Ala-Trp-Leu-Val-Arg-Gly-Arg-Gly
	semaglutide	polypeptide	H-His-Ala-Glu-Gly-Thr-Phe-Thr-Ser-Asp-Val-Ser-Ser-Tyr-Leu-Glu-Gly-Gln-Ala-Ala-Lys-Glu-Phe-Ile-Ala-Trp-Leu-Val-Arg-Gly-Arg-Gly-OH
	Orforglipron (LY3502970/OWL833)	partial, G protein-biased agonist	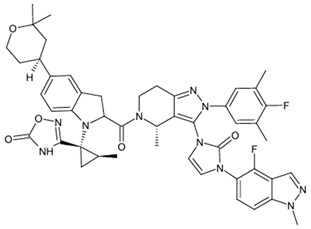
	danuglipron	agonist	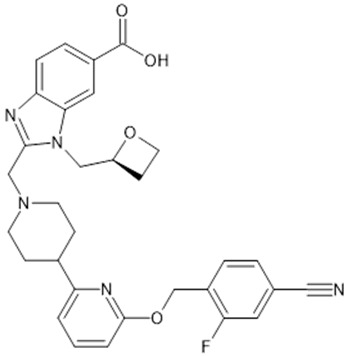
ADRB1,2,3	Norepinephrine	endogenous agonist	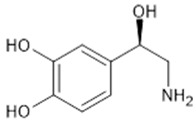
	epinephrine	endogenous agonist	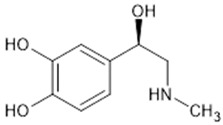
	Isoproterenol	nonselective ADRB antagonist	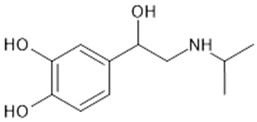
	Propranolol	nonselective ADRB antagonist	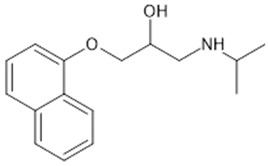
ADRB1	CGP20712A	selective ADRB1 antagonist	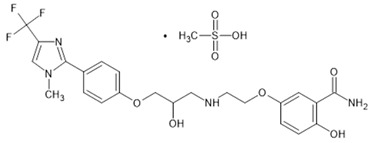
	Atenolol	selective ADRB1 antagonist	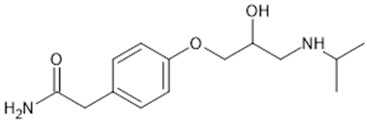
ADRB2	Formoterol	selective ADRB2 agonist	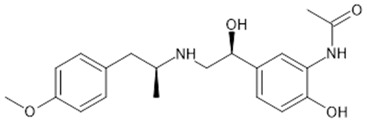
	Salbutamol	selective ADRB2 agonist	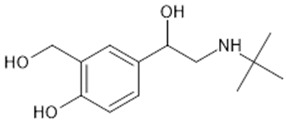
	Fenoterol	selective ADRB2 agonist	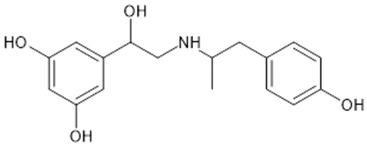
ADRB3	Mirabegron	selective ADRB3 agonist	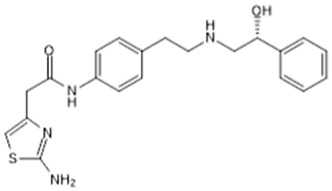
	CL-316,243	selective ADRB3 agonist	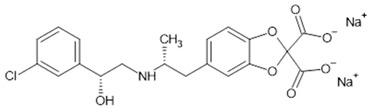

## Data Availability

Not applicable.
